# The Role of Epithelial Cell Adhesion Molecule Cancer Stem Cell Marker in Evaluation of Hepatocellular Carcinoma

**DOI:** 10.3390/medicina60060915

**Published:** 2024-05-30

**Authors:** Marwa A. El-Kholy, Shimaa S. Abu-Seadah, Abdulkarim Hasan, Mohammed E. A. Elhussiny, Mohammed S. Abdelwahed, Mehenaz Hanbazazh, Abdulhadi Samman, Saeed A. Alrashdi, Zaky F. Rashed, Diaa Ashmawy, Alyaa E. Othman, Mohamed F. Abdelaleem, Amany I. A. Abo-Saif, Rania R. Abdel-Maqsoud, Samah M. Attiah, Eissa Saeed Assiri, Mohamed Nasr, Khadiga Ahmed Ismail, Diana Z. Saad, Marwa M. El-Mosely

**Affiliations:** 1 Pathology Department, Faculty of Medicine for Girls, Al-Azhar University, Cairo 11884, Egypt; 2 Pathology Department, Faculty of Medicine, Al-Azhar University, Cairo 11884, Egypt; 3 General Medicine Practice Program, Histology Department, Batterjee Medical Collage, Aseer 61421, Saudi Arabia; 4 Histology Department, Faculty of Medicine, Al-Azhar University, Cairo 11884, Egypt; 5 Pathology Department, Faculty of Medicine, University of Jeddah, Jeddah 23218, Saudi Arabia; 6 Laboratory Department, Al-Mezailef General Hospital, Ministry of Health, Al-Mezailef 21912, Saudi Arabia; 7 Anesthesia Department, College of Applied Sciences, AlMaarefa University, Riyadh 71666, Saudi Arabia; 8 Anesthesia, Intensive Care and Pain Management Department, Faculty of Medicine, Al-Azhar University, Cairo 11884, Egypt; 9 Pathology Department, Faculty of Medicine, Al-Azhar University, Damietta 34517, Egypt; 10 Infectious Diseases Department, Faculty of Medicine, Suez Canal University, Ismailia 41522, Egypt; 11 Preventive Medicine, Ministry of Health, Cairo 51222, Egypt; 12 Laboratory Department, Aseer Central Hospital, Ministry of Health, Abha 62523, Saudi Arabia; 13 Department of Clinical Laboratory Sciences, College of Applied Medical Sciences, Taif University, Taif 21944, Saudi Arabia; 14 Pathology Department, Faculty of Medicine, Ain Shams University, Cairo 11566, Egypt; 15 Pathology Department, Faculty of Medicine, Zagazig University, Zagazig 44519, Egypt

**Keywords:** cancer stem cells, Epithelial cell adhesion molecule, EpCAM, hepatocellular carcinoma, immunohistochemistry

## Abstract

*Background and Objectives:* Hepatocellular carcinoma (HCC) is a prevalent form of malignancy that is characterized by high mortality rates and prognosis that remain suboptimal, largely due to treatment resistance mechanisms. Recent studies have implicated cancer stem cells (CSCs), particularly those expressing epithelial cell adhesion molecule (EpCAM), in HCC progression and resistance. In the present study, we sought to assess EpCAM expression in HCC patients and its correlation with various clinicopathological parameters. *Materials and Methods:* Tissue samples from 42 HCC patients were subjected to immunohistochemical staining to evaluate EpCAM expression. Clinicopathological data were obtained including the size, grade and stage of tumors, vascular invasion status, alpha-fetoprotein levels, and cirrhosis status. The Chi square and Fisher’s exact tests were employed to assess the association between categorical groups. Independent Student-t test or Mann–Whitney U test was used to investigate the association between continuous patient characteristics and survival. *Results:* Immunohistochemical analysis revealed EpCAM expression in 52.5% of HCC cases. EpCAM-positive tumors exhibited characteristics indicative of aggressive disease, including larger tumor sizes (*p* = 0.006), greater tumor multiplicity (*p* = 0.004), higher grades (*p* = 0.002), more advanced stages (*p* = 0.003), vascular invasion (*p* = 0.023), elevated alpha-fetoprotein levels (*p* = 0.013), and cirrhosis (*p* = 0.052). Survival analysis demonstrated that EpCAM expression was significantly associated with lower overall rates of survival and higher rates of recurrence in HCC patients. *Conclusions:* Our findings suggest that EpCAM expression may serve as a prognostic biomarker for HCC with a potential role in patient management. Targeting EpCAM-positive CSCs may represent a promising approach to overcome treatment resistance and improve clinical outcomes in HCC. However, further investigation into the molecular mechanisms underlying EpCAM’s role in HCC progression is warranted to facilitate the development of personalized therapeutic interventions.

## 1. Introduction

In the year 2020, 905,677 cases of primary liver cancer were reported worldwide, resulting in 830,180 deaths, making this the sixth-most prevalent cancer type, and the second-leading cause of cancer mortality among males [1]. Approximately 90% of all primary liver tumors are hepatocellular carcinomas (HCCs). Roughly 85% of individuals with cirrhosis will develop hepatocellular carcinoma. Currently, HCC ranks as the sixth-most prevalent cause of cancer globally [2,3]. Most people respond poorly to traditional therapies like radiation and chemotherapy, and this may be because cancer stem cells (CSCs) are present in the patient population [4].

A tiny subset of tumor cells exhibit stem cell characteristics such as self-renewal and widespread proliferation; these are known as CSCs [5], and they have been associated with enhanced DNA repair and inhibition of apoptosis [6].

For liver cancer, several cancer stem cell markers have been found, including EpCAM, CD133, CD90, and CD13 [7].

Studies of EpCAM have indicated that it may play roles in cancer stemness, cell proliferation, metabolism, angiogenesis, metastasis, resistance to chemotherapy and radiation, and immunomodulation [8,9].

Most human epithelial carcinomas, including those of the liver, breast, colon, prostate, and head and neck regions, have overexpressed EpCAM. Moreover, several human carcinomas are now treated with immunotherapy using EpCAM as a target [10].

As a tumor grows, EpCAM interacts with numerous key signaling pathways, including p53, TGF-β/SMAD, EpEX/EGFR, PI3K/AKT/mTOR, and Wnt/β-catenin, to alter the biology of cancer cells [11,12].

Yamashita et al. showed that, even with standard chemotherapy, EpCAM-positive HCC is characterized by poor prognosis and a high probability of tumor recurrence [13].

However, tumors may be eliminated without recurrence by targeting EpCAM with certain monoclonal antibodies, by gene silencing, and by blocking Wnt/β-catenin signaling [14].

## 2. Materials and Methods

### 2.1. Tissue Samples

Paraffin blocks of 42 liver resection (LR) cases for hepatocellular carcinoma dating from between January 2017 and April 2022 were obtained from the surgical archives of the Histopathology Department of Al-Azhar University Hospital after approval was granted by the research ethical committee. Cases were selected if there was sufficient specimen material on the paraffin blocks and good clinicopathological data in hospital records relating to the age and gender of patients, histological grades and stages, the diversity and size of tumors, vascular invasion, associated cirrhosis or viral hepatitis, α-fetoprotein (AFP) levels, microvessel invasion, intrahepatic metastasis, and follow-up data. Pathological staging was determined according to the 8th edition of the Cancer Staging Manual published by the American Joint Committee on Cancer. In all cirrhotic liver cases, hepatitis virus infection (HCV and HBV) was additionally present.

The time period between the first surgical intervention and the date of the last follow-up (or the patient’s death from HCC) was termed the follow-up period. To reassess diagnoses, representative samples were stained with hematoxylin and eosin. Histological grades of hepatocellular carcinoma were determined using the Edmondson–Steiner grading system [15]. Exclusion criteria included cases for which tissue blocks were unavailable in our institution as well as cases lacking related clinical data.

### 2.2. Immunohistochemistry

Immunohistochemical (IHC) staining was performed using the standard streptavidin–biotin–peroxidase complex (ABC) method (DakoCytomation, CA, USA). Tissue samples of 3–5 microns thickness were prepared using 10% formalin-fixed, paraffin-embedded liver specimens of representative tumor areas; these were then deparaffinized in xylene and then rehydrated in graded alcohols. Sections were boiled in citrate buffer (pH 6.0) for 20 min, followed by rinsing in distal water and washing in phosphate buffer saline. Endogenous peroxidase activity was blocked by incubating the sections for 15 min with 0.3% hydrogen peroxide in absolute methanol. The slides were then incubated overnight with anti-EpCAM antibody (Mouse, B302–323/A3, Abcam, Cambridge, UK, 1:200). After washing with PBS, the slides were incubated with multilink secondary antibody, after which goat anti-mouse streptavidin–biotin–peroxidase reagent was applied for 30 min (Dako, Japan). Finally, sections were incubated with diaminobenzidine, counter-stained with hematoxylin, and then cleared and mounted.

### 2.3. Interpretation of the Staining

The expression of EpCAM was examined based on the intensity of staining and the percentage of positive cells. The IHC results were assessed using the extent of cell staining, which ranged from 0% to 100%. When no positive cells were found, the degree of positivity was given a score of 0; other outcomes were semi-quantitatively evaluated as follows: negative, <5%; weak (1+), 5–30%; moderate (2+), 30–60%; or strong (3+), >60%. If more than 10% of the cells had a final staining score that was moderate or strong, the expression of EpCAM and other stemness-related markers was deemed positive [16]. The slide examination was conducted on-site by expert histopathologists with similar levels of experience, or by remote examination telepathologically.

### 2.4. Positive and Negative Controls

As a negative control, the main antibody was left out. The bile duct epithelium served as EpCAM’s internal positive control. Positive and negative controls were implemented concurrently.

### 2.5. Statistical Analysis

Data were collected and coded using a Microsoft Excel spreadsheet. The Statistical Package for Social Science (SPSS, IBM Inc., Armonk, NY, USA, Windows version 25) was used to conduct all statistical analyses. The Shapiro–Wilk test for data normality was used. Normally distributed continuous data were presented as mean and standard deviation (SD), while medians and interquartile ranges (IQRs) were used to present non-normally distributed data. Categorical variables were presented as frequencies and percentages. Chi-squared tests and Fisher’s exact tests were employed to assess the association between categorical groups. An independent Student *t*-test or a Mann–Whitney U test was used to investigate the association between continuous patient characteristics and survival. *p*-values of ≤0.05 were deemed to be significant.

## 3. Results

**Figure 1:**
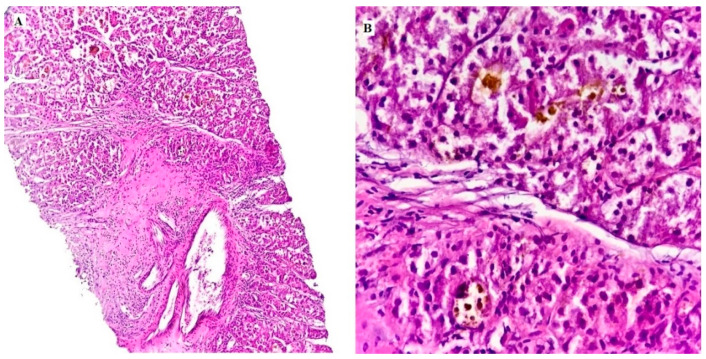
A histological picture of a case of HCC showing hepatocytes with nuclear atypia and pigments (**A**): low power image, H&E, 40×) & (**B**): High power, H&E, 200× original magnification).

The study included a total of 42 patients with a mean age of 50.1 years and a median tumor size of 5.75 cm. Most patients were female (62%), and the prevalence of tumor multiplicity was 19%. High-grade tumors were more frequent than low-grade tumors (59.5% vs. 40.5%) (Figure 1). In terms of stages of disease, the greatest number of patients were at stage I (45.2%), followed by stage III (33.3%). Most patients (74%) had associated cirrhosis but no vascular invasion. Regarding AFP levels, 64% of the patients had levels greater than 100 ng/mL. Finally, EpCAM expression was almost evenly distributed, with 52.5% of patients expressing this marker (Table 1).

### 3.1. EpCAM Expression in Different Studied Cases

**Figure 2:**
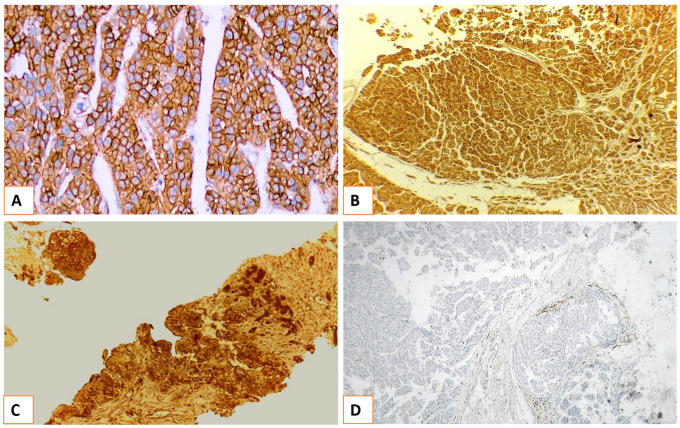
Immunohistochemical staining of resected cases of HCC specimens for epithelial cell adhesion molecule (EpCAM). (**A**): HCC showed positive (EpCAM) staining (200×). (**B**): Specimen positive for EpCAM expression in large-sized nodule (100×). (**C**): Specimen positive for EpCAM expression in multiple tumor nodules (100×). (**D**): specimen negative for EpCAM expression in low-grade HCC (100× original magnification).

Significant differences were observed between EpCAM-positive and EpCAM-negative groups across various clinical and pathological characteristics (Figure 2). Patients with EpCAM-positive tumors were more likely to have larger tumor sizes (>5 cm) than those with EpCAM-negative tumors, with 71% of EpCAM-positive patients having tumor sizes greater than 5 cm, compared with 29% of patients in the EpCAM-negative group (*p* = 0.006). Notably, all patients (100%) with multiple tumors were in the EpCAM-positive group (*p* = 0.004). Additionally, a higher percentage of patients in the EpCAM-positive group had high-grade tumors (72%), compared with patients in the EpCAM-negative group (28%) (*p* = 0.002). Differences were also observed with respect to the stages of patients’ cancers, with a notably higher proportion of stage III patients in the EpCAM-positive group (86%), compared with the EpCAM-negative group (14%) (*p* = 0.003). Vascular invasion was more prevalent in patients with EpCAM-positive tumors, with 82% of these patients exhibiting vascular invasion, compared with just 18% of patients in the EpCAM-negative group (*p* = 0.023). Regarding patients who had associated cirrhosis, we obtained statistically significant values (*p* = 0.052) for EpCAM positivity, with 61% of cirrhotic patients showing positive expression, compared with 27% of non-cirrhotic patients. Furthermore, patients with EpCAM-positive tumors were significantly more likely to have an AFP level which was higher than 100 ng/mL (67%) than patients with EpCAM-negative tumors (33%) (*p* = 0.013) (Table 2).

### 3.2. Correlations between Survival and Different Clinicopathological Parameters

A comparison between survivors and non-survivors with respect to various clinicopathological parameters is presented in Table 3. Among non-survivors, a significantly higher prevalence of higher-stage cancer was evident, with all such cases being stage III (*p* = 0.006). Below the level of statistical significance, the following results were also obtained: all non-survivors were found to have high-grade tumors, with tumor sizes greater than 5 cm; all non-survivors also had associated cirrhosis and a high AFP level of above 100 ng/mL; in three cases, non-survivors showed vascular invasion (60%, *p* = 0.134); in four cases, non-survivors were EpCAM positive (80%, *p* = 0.355). No significant differences between survivor and non-survivor groups were found with respect to patient age, sex, or tumor multiplicity.

### 3.3. Correlations between Recurrence and Different Clinicopathological Parameters

A comparison between the group of patients who experienced a recurrence of the disease and the group in which the disease did not recur is presented in Table 4. The mean age was significantly higher in the recurrent group (62.2 years), compared with the non-recurrent group (40.7 years; *p* = 0.003). Recurrent cases showed significantly higher prevalences of high stages (all cases were stage III, *p* < 0.001), high grades (all cases were high grade, *p* = 0.006), large tumor sizes (all cases were >5 cm, *p* = 0.005), vascular invasion (all cases showed vascular invasion, *p* < 0.001), AFP levels (all cases showed high levels of AFP > 100 ng/mL, *p* = 0.016), associated cirrhosis (all cases showed associated cirrhosis, *p* = 0.079), and EpCAM expression (all cases showed positive EpCAM expression, *p* = 0.002). In addition, tumor multiplicity was present in all recurrent cases (100%) but absent in all non-recurrent cases (*p* < 0.001).

## 4. Discussion

HCC is among the leading causes of cancer-related mortality worldwide [17], with males twice as likely as females to be diagnosed with the disease [18]. HCC is highly resistant to current chemotherapeutic treatments, and the survival rate for the disease is low [19]. Overall, the increasing incidence of liver cancer may be seen as placing a significant burden on human societies [20]. However, the discovery of novel biomarkers may lead to improvements in HCC survival rates. Such biomarkers may be used to predict outcomes, enabling clinical practitioners to select better treatment options and prevent needless side effects, in HCC patients [17].

It has been shown that HCC contains CSCs; these are a small but distinct minority of cells that consistently display stem cell characteristics such as self-renewal, cell proliferation, and differentiation [21]. One surface marker of CSCs has been identified as the EpCAM [22]. Furthermore, poor HCC prognosis has been associated with EpCAM expression, indicating that EpCAM may be a useful biomarker for risk classification [21,23]. Therefore, its identification in individuals with HCC may be a significant prognostic factor [24]. EpCAM-negative HCC is characterized by short telomerase length and limited proliferation [25]. However, the extent to which the intensity and spatial distribution of intratumoral EpCAM expression influences the spread and local aggressiveness of metastasis remains unknown [26].

The transmembrane protein EpCAM is considered to have multiple functions; in cancer cells, it is involved in the control of stemness, cell adhesion, proliferation, migration, and epithelial-to-mesenchymal transition. To carry out these tasks, EpCAM is essential for both intra- and intercellular communication as a whole molecule and, after controlled intramembrane proteolysis, for producing extracellular and intracellular fragments that are functionally active [27].

Overexpression of EpCAM has been detected in various human carcinomas, including cancer of the breast [28], pancreas [29], and liver [13]. Such overexpression makes EpCAM a novel molecular target for oncological therapy. In addition, in epithelial ovarian cancer, overexpression of EpCAM has been associated with a higher risk of tumor malignancy, further suggesting that EpCAM expression might serve as a molecular therapeutic target for advanced-stage epithelial ovarian cancer and as a possible biomarker for monitoring the disease’s progression [30].

EpCAM has also emerged as a metric for the ability of circulating tumor cells (CTCs) to metastasize and thus serve as a marker for the epithelial state of primary and systemic tumor cells. As a result, EpCAM’s potential as a target and prognostic marker for primary and systemic tumor cells has been confirmed [29].

In the present study, a total of 42 cases were considered. The median tumor size was 5.75 cm and the mean patient age was 50.1 years. Sixty-two percent of the patients were female, and nineteen percent of patients had multiple tumors. A higher percentage of patients had high-grade tumors than low-grade tumors (59.5% vs. 40.5%), and the greatest number of patients had stage I illness (45.2%), followed by stage III (33.3%). Most patients (74%) had concomitant cirrhosis but no vascular invasion. Sixty-four percent of the patients had AFP levels higher than 100 ng/mL. Out of all patients studied, 52.5% had EpCAM expression, which was nearly uniformly distributed.

Our pathological data revealed that expression of the EpCAM marker was observed in 22 (52.4%) out of 42 cases of hepatocellular carcinoma; this result is in agreement with the findings of other studies by Yamashita et al., 2008 [13], Kim et al., 2011 [30], and Shan et al., 2010 [31], who found that between 15.9% and 48.7% of all hepatocellular carcinomas expressed EpCAM.

In the present study, we found that, between the EpCAM-positive and EpCAM-negative groups, there were notable variations with respect to several clinical and pathological traits. Individuals with EpCAM-positive tumors were more likely to have larger tumor sizes (>5 cm) than individuals with EpCAM-negative tumors. Among EpCAM-positive patients, 71% had tumor sizes greater than 5 cm, compared with 29% in the EpCAM-negative group (*p* = 0.006). This result is contrary to the findings of Lima et al., 2018 [7] who reported that, among 35 small-size cases (<2 cm), EpCAM expression was detected in 54% of tumors, suggesting that this molecule plays an important role in early stages of tumorogenesis due to its stem cell properties. In the present study, none of the patients with multiple tumors were in the EpCAM-negative group (*p* = 0.004), while all patients (100%) with multiple tumors were in the EpCAM-positive group. This result is in line with the findings of Krause et al., 2020 [26], who reported that EpCAM expression (homogeneous distribution) was significantly associated with higher levels of serum AFP (*p*= 0.03), and thus confirmed the previous findings of Bae et al., 2012 [32] and Yamashita et al., 2008 [13]. Regarding pathological stages and histological grades, we found that higher EpCAM expression was associated with high stages and high grades (*p* = 0.003) and (*p* = 0.002), respectively). Previously, Xu et al., 2014 [23] studied HCC patients with high EpCAM expression and found that patients with advanced TNM stages and high AFP levels were more likely to have aggressive clinical characteristics, including greater relapse rates, in line with the results of the present study. In addition, a meta-analysis conducted by Liu et al. in 2015 [33], demonstrated that EpCAM expression was associated with poor differentiation of HCCs.

In the present study, we also found that increased EpCAM expression was associated with increased AFP levels and vascular invasion (*p* = 0.013) and (*p* = 0.023), respectively). Similarly, Abdelgawad, 2020 [24] detected higher serum levels of AFP among EpCAM-positive cases, compared with EpCAM negative cases (*p* = 0.022) [24], with five out of thirteen (38%) EpCAM-positive cases having AFP levels > 400 ng/dl. Yamashita et al., 2013 [34] found that EpCAM-positive CTCs were associated with poor prognoses and unfavorable criteria such as the presence of vascular invasion, high levels of AFP, and poor differentiation. The findings of Kelley and Venook, 2013 [35] suggested a potential role for CTCs in the prognostic stratification of HCC patients and decision-making with regard to treatment, both of which may be seen as challenging because of the great prognostic heterogeneity of this disease. Tsuchiya et al., 2019, reported that 10–20% of cancer cells in primary tumors expressed NCAM (NCAM2+) and 5–10% expressed EpCAM (EpCAM1+), indicating that cancer cells positive for both markers exhibited more extensive vascular invasion, compared with cancer cells negative for HPC markers [36].

Clinicians ought to identify patients with resected HCC who are at a higher risk of recurrence following treatment. This is crucial for determining whether additional medications or further follow-up are required, as prognostic predictive value is essential [16].

In the present study, we carried out a comparison of survivors and non-survivors among the 42 patients in our study population. We found that the prevalence of high stages was substantially higher in non-survivors (all cases were stage III, *p* = 0.006). In addition, all of the non-survivors had high-grade tumors measuring more than 5 cm, along with concomitant cirrhosis and high AFP levels of approximately 100 ng/mL; however, these results were not statistically significant. Vascular invasion was present in three non-survivor cases (60%, *p* = 0.134), and four non-survivor cases (80%, *p* = 0.355) which tested positive for EpCAM. There were no significant differences between the survivor and non-survivor groups in terms of age, sex, or tumor multiplicity. We also compared groups of patients amongst whom the disease either recurred or did not recur. In comparison with the non-recurrent group (40.7 years; *p* = 0.003), the mean age in the recurrent group was significantly higher (62.2 years). The recurrent cases exhibited a significantly higher prevalence of high stages (all cases were stage III, *p* < 0.001), high grades (all cases were high grade, *p* = 0.006), large sizes (all cases were 5 cm, *p* = 0.005), high AFP levels (all cases showed a level of AFP >100 ng/mL, *p* = 0.016), associated cirrhosis (all cases showed associated cirrhosis, *p* = 0.079), and EpCAM expression (all cases showed positive EpCAM expression, *p* = 0.002). Tumor multiplicity was exhibited in all patients in whom disease recurred (100%) but was wholly absent amongst non-recurrent patients (*p* < 0.001). The IHC expression of EpCAM was previously confirmed by Noh et al., 2018 [16]. In the present study, this was linked to a lower overall survival rate and an increased chance of recurrence in patients with HCC. In addition, high levels of blood AFP and positive EpCAM expression were linked to fast recurrence following surgical resection. These findings imply that a preoperative biopsy may be used to forecast a patient’s prognosis and that the study of specimens removed during surgery may be of value in this regard.

Another study by Zhou et al. in 2016 [37], showed an association between the preoperative presence of EpCAM-expressing CTCs and T-regulatory cell levels with HCC tumor recurrence after resection. Similarly, Schulze et al., 2013 [38] found that patients with EpCAM-positive CTCs had significantly reduced overall survival rates, in comparison with patients without these cells (*p* = 0.017), and that the presence of CTCs was correlated with high levels of serum AFP (*p* = 0.050). Moreover, von Felden et al., 2017 [14], reported a correlation between EpCAM-positive CTCs and high recurrence rates (HR = 2.3, *p* = 0.027), with shorter periods of recurrence-free survival among patients who underwent curative resection for HCC (5.0 ± 1.5 vs. 12.0 ± 2.6 months, *p* = 0.039).

In the present study, we discovered that EpCAM expression was a predictor of low survival rates and poor recurrence prognoses in patients who underwent surgical resection for HCC, even after controlling for clinicopathological prognostic factors. We believe that, for patients with HCC who undergo hepatic resection and percutaneous biopsy, EpCAM immunohistochemical expression may be utilized to predict prognosis.

We wholeheartedly support the conclusions of earlier studies in which EpCAM was shown to be a significant biomarker and prognostic factor for HCC. This chemical is thought to be a biomarker for CTC and CSC detection; as such, it offers potentially novel methods for diagnosis and prognosis [31,39].

Vasanthakumar et al., 2017 stated that EpCAM can be used as a cancer stem cell marker and as a potential therapeutic target for EpCAM-positive tumors. Targeting EpCAM can eradicate tumors without any relapse, whether this is achieved by gene silencing, inhibition of Wnt/β-catenin signaling, vaccination, nanomedicinal approaches, or the use of specific monoclonal antibodies, [4]. Gene silencing is a technique used to knock down a desired gene by using RNAi (siRNA) to inhibit a particular gene function. EpCAM is a marker of many carcinomas and cancer stem cells involved in a variety of functions such as cell proliferation, cell migration, invasion, metastasis, chemoresistance, and tumor relapse. So, silencing the EpCAM gene can help conventional chemotherapy to work more effectively without any influence of cancer stem cell activity [40]

This study has several limitations including a low total number of cases and a lack of evaluation of peritumoral marker expression. We were also unable to assess the relation of expression with the underlying etiology. We recommend further studies on patients from multiple centers with the inclusion of ancillary genetic testing.

## 5. Conclusions

The results of our analysis, taken together with the findings of previous studies, indicate that the overexpression of EpCAM may be connected to clinicopathological traits of HCC, including poor differentiation and elevated AFP levels. Gene silencing is a technique used to knock down a target gene by employing RNA interference (siRNA) to limit the activity of specific genes. Knockdown of EpCAM has been shown to reduce proliferation and spheroid formation in several EpCAM-positive cell lines and enhance chemo- and radiosensitivity. However, more clinical and experimental studies are needed to determine the likely molecular pathways of EpCAM for HCC.

## Figures and Tables

**Table 1:** Demographic and tumor characteristics of all patients.

Characteristics	N (%)
N = 42	
Age, mean (SD)	50.1 (13)
Tumor Size, median (IQR)	5.75 (3.38)
Size	
>5	24 (57%)
≤5	18 (43%)
Sex	
Male	16 (38%)
Female	26 (62%)
Tumor Multiplicity	
Yes	8 (19%)
No	34 (81%)
Grade	
Low	17 (40.5%)
High	25 (59.5%)
Stage	
I	19 (45.2%)
II	9 (21.4%)
III	14 (33.3%)
Vascular Invasion	
Present	11 (26%)
Absent	31 (74%)
Associated Cirrhosis	
Yes	31 (74%)
No	11 (26%)
AFP (ng/mL)	
>100	27 (64%)
≤100	15 (36%)
EpCAM expression	
Positive	22 (52.5%)
Negative	20 (47.5%)

EpCAM: Epithelial cell adhesion molecule, AFP: Alpha-fetoprotein, SD: Standard deviation; IQR, interquartile range.

**Table 2:** Comparison between EpCAM positive and negative.

**Characteristics**	**EpCAM Positive**	**EpCAM Negative**	**Total**	***p*-Value**
N	22	20	42 (100%)	
Age, mean (SD)	52 (12.7)	47.4 (13.2)		0.202
Tumor size, median (IQR)	6 (1.88)	5 (3.25)		0.061
Size				0.006
>5	17 (71%)	7 (29%)	24 (100%)	
≤5	5 (28%)	13 (72%)	18 (100%)	
Sex				0.694
Male	9 (56%)	7 (44%)	16 (100%)	
Female	13 (50%)	13 (50%)	26 (100%)	
Tumor Multiplicity				0.004
Yes	8 (100%)	0 (0%)	8 (100%)	
No	14 (41%)	20 (59%)	34 (100%)	
Grade				0.002
Low	4 (23.5%)	13 (76.5%)	17 (100%)	
High	18 (72%)	7 (28%)	25 (100%)	
Stage				0.003
I	5 (26%)	14 (74%)	19 (100%)	
II	5 (55.5%)	4 (44.5%)	9 (100%)	
III	12 (86%)	2 (14%)	14 (100%)	
Vascular Invasion				0.023
Present	9 (82%)	2 (18%)	11 (100%)	
Absent	13 (42%)	18 (58%)	31 (100%)	
Associated Cirrhosis				0.052
Yes	19 (61%)	12 (39%)	31 (100%)	
No	3 (27%)	8 (73%)	11 (100%)	
AFP (ng/mL)				0.013
>100	18 (67%)	9 (33%)	27 (100%)	
≤100	4 (27%)	11 (73%)	15 (100%)	

EpCAM: Epithelial cell adhesion molecule, AFP: Alpha-fetoprotein, SD: Standard deviation; IQR, interquartile range.

**Table 3:** Comparison between survivors and non-survivors.

**Characteristic**	**Survivors**	**Non-Survivors**	**Total**	***p*-Value**
N	33	5	38 (100%)	
Age, mean (SD)	50.8 (14.1)	53.4 (6.6)		0.685
Tumor size, median (IQR)	5.5 (4)	6 (1.5)		0.557
Size				0.061
>5	17 (77%)	5 (23%)	22 (100%)	
≤5	16 (100%)	0 (0%)	16 (100%)	
Sex				0.337
Male	11 (78.5%)	3 (21.5%)	14 (100%)	
Female	22 (92%)	2 (8%)	24 (100%)	
Tumor Multiplicity				0.279
Yes	6 (75%)	2 (25%)	8 (100%)	
No	27 (90%)	3 (10%)	30 (100%)	
Grade				0.136
Low	15 (100%)	0 (0%)	15 (100%)	
High	18 (78%)	5 (22%)	23 (100%)	
Stage				0.006
I	17 (100%)	0 (0%)	17 (100%)	
II	7 (100%)	0 (0%)	7 (100%)	
III	9 (64%)	5 (36%)	14 (100%)	
Vascular Invasion				0.134
Present	8 (73%)	3 (27%)	11 (100%)	
Absent	25 (92.5%)	2 (7.5%)	27 (100%)	
Associated Cirrhosis				0.298
Yes	23 (82%)	5 (18%)	28 (100%)	
No	10 (100%)	0 (0%)	10 (100%)	
AFP (ng/mL)				0.144
>100	20 (80%)	5 (20%)	25 (100%)	
≤100	13 (100%)	0 (0%)	13 (100%)	
EpCAM expression				0.355
Positive	17 (81%)	4 (19%)	21 (100%)	
Negative	16 (94%)	1 (6%)	17 (100%)	

EpCAM: Epithelial cell adhesion molecule, AFP: Alpha-fetoprotein, SD: Standard deviation; IQR, interquartile range.

**Table 4:** Comparison between recurrent and non-recurrent.

**Characteristic**	**Recurrent**	**Non-Recurrent**	**Total**	***p*-Value**
N	9	29	38 (100%)	
Age, mean (SD)	62.2 (8.4)	40.7 (12.7)		0.003
Tumor size, median (IQR)	6 (1.25)	5.25 (4.88)		0.40
Size				0.005
>5	9 (41%)	13 (59%)	22 (100%)	
≤5	0 (0%)	16 (100%)	16 (100%)	
Sex				1.00
Male	3 (21.5%)	11 (78.5%)	14 (100%)	
Female	6 (25%)	18 (75%)	24 (100%)	
Tumor Multiplicity				<0.001
Yes	8 (100%)	0 (0%)	8 (100%)	
No	1 (3%)	29 (97%)	30 (100%)	
Grade				0.006
Low	0 (0%)	15 (100%)	15 (100%)	
High	9 (39%)	14 (61%)	23 (100%)	
Stage				<0.001
I	0 (0%)	17 (100%)	17 (100%)	
II	0 (0%)	7 (100%)	7 (100%)	
III	9 (64%)	5 (36%)	14 (100%)	
Vascular Invasion				<0.001
Present	9 (82%)	2 (18%)	11 (100%)	
Absent	0 (0%)	27 (100%)	27 (100%)	
Associated Cirrhosis				0.079
Yes	9 (32%)	19 (68%)	28 (100%)	
No	0 (0%)	10 (100%)	10 (100%)	
AFP (ng/mL)				0.016
>100	9 (36%)	16 (64%)	25 (100%)	
≤100	0 (0%)	13 (100%)	13 (100%)	
EpCAM expression				0.002
Positive	9 (43%)	12 (57%)	21 (100%)	
Negative	0 (0%)	17 (100%)	17 (100%)	

EpCAM: Epithelial cell adhesion molecule, AFP: Alpha-fetoprotein, SD: Standard deviation; IQR, interquartile range.

## Data Availability

The original contributions presented in the study are included in the article, further inquiries can be directed to the corresponding author/s.
